# Impact of the Healthy Foods North nutrition intervention program on Inuit and Inuvialuit food consumption and preparation methods in Canadian Arctic communities

**DOI:** 10.1186/1475-2891-13-68

**Published:** 2014-07-04

**Authors:** Fariba Kolahdooz, Mohammadreza Pakseresht, Erin Mead, Lindsay Beck, André Corriveau, Sangita Sharma

**Affiliations:** 1 Aboriginal and Global Health Research Group, Department of Medicine, Faculty of Medicine and Dentistry, University of Alberta, Unit 5-10, University Terrace, 8303-112 Street, Edmonton, AB T6G 2T4, Canada; 2 Department of Health, Behavior and Society, Bloomberg School of Public Health, Johns Hopkins University, Baltimore, MD, USA; 3 Office of the Chief Public Health Officer, Department of Health and Social Services, Government of the Northwest Territories, 6th Floor, Centre Square Tower, Box 1320, Yellowknife, NT X1A 2L9, Canada

**Keywords:** Healthy Foods North, Inuit, Inuvialuit, Food consumption, Food preparation, Arctic, Dietary intervention

## Abstract

**Background:**

The 12-month Healthy Foods North intervention program was developed to improve diet among Inuit and Inuvialuit living in Arctic Canada and assess the impact of the intervention established for the communities.

**Methods:**

A quasi-experimental study randomly selected men and women (≥19 years of age) in six remote communities in Nunavut and the Northwest Territories. Validated quantitative food frequency and adult impact questionnaires were used. Four communities received the intervention and two communities served as delayed intervention controls. Pre- and post-intervention changes in frequency of/total intake of de-promoted food groups and healthiness of cooking methods were determined. The impact of the intervention was assessed using analysis of covariance (ANCOVA).

**Results:**

Post-intervention data were analysed in the intervention (n = 221) and control (n = 111) communities, with participant retention rates of 91% for Nunavut and 83% for the Northwest Territories. There was a significant decrease in de-promoted foods, such as high fat meats (−27.9 g) and high fat dairy products (−19.8 g) among intervention communities (all *p ≤* 0.05). The use of healthier preparation methods significantly increased (14.7%) in intervention communities relative to control communities.

**Conclusions:**

This study highlights the importance of using a community-based, multi-institutional nutrition intervention program to decrease the consumption of unhealthy foods and the use of unhealthy food preparation methods.

## Background

Approximately 30% of Inuit and 35% of Inuvialuit over the age of 15 years reported having at least one chronic health condition in 2008 [1]. Additionally, about 57% of the population residing in the Northwest Territories is overweight or obese, compared to 54% of the national Canadian average [2]. Overall, Indigenous people in Canada have a life expectancy 8 to 13 years lower than non-Indigenous Canadians [3,4], which may contribute to this populations’ relatively high health care costs (approximately 1.8-2.2 times the Canadian average) [5].

The lifestyle and diet changes experienced by Indigenous peoples during the last decades influence diet quality, which affects the prevalence of nutrition-related chronic diseases (e.g. hypertension, diabetes, obesity, and some forms of cancer) in Canadian Indigenous populations residing in Nunavut (NU) and the Northwest Territories (NWT) [6-10]. Several studies of Inuit and Inuvialuit populations have documented rapid socio-economic and cultural transitions related to acculturation which have ultimately led to a shift away from traditional diets and procurement practices towards increased dependence on non-nutrient-dense, store-bought foods [10-13]. Populations residing in NU and the NWT are further challenged by their remote location [13] and the increasing cost of food [14], creating reliance on inexpensive non-perishable processed foods that are usually non-nutrient-dense [15].

Recent assessments in Inuit and Inuvialuit adults found that dietary fiber, calcium, folate, and vitamins A and E (and vitamin D among women) were below the recommendations in 60-100% of participants [16,17]. In addition, recent studies found a high prevalence of preparation methods that add fat to foods (e.g. frying with lard) [18,19]; these preparation methods are determinants of fat intake [20,21] and risk factors for impaired glucose tolerance [22] among Inuit and Inuvialuit populations.

Health promotion programs that concentrate on healthy eating for the reduction of chronic disease should use a comprehensive approach that combines individual, organizational and policy levels in order to effectively address the multilevel risk factors [6]. Furthermore, store-based environmental interventions that integrate behavior change strategies have proven effective in improving diet with other Indigenous and low-income populations [23-25]. Combined environmental and behavioral approaches have been shown to be one of the most promising ways to improve diet and reduce risk of chronic disease [26].

Healthy Foods North (HFN) was an evidence-based intervention program designed specifically for Inuit and Inuvialuit populations to reduce the risk of chronic disease by improving diet and increasing physical activity. HFN combined behavioral and environmental strategies through community-based activities, multi-institutional partnerships, and point of purchase to increase the availability, accessibility and visibility of healthy foods as well as opportunities for physical activity. HFN was tailored to build on the strengths and meet the specific needs of the communities through culturally appropriate programming [24,27-29]. Key elements of HFN included the promotion of healthier food preparation methods, the multitude of benefits related to traditional foods, and healthier options in stores [13,28]. These traditions included food sharing, survival from the land and respect for food [30].

The purpose of this study was to evaluate the 12 month HFN intervention program by: 1) determining pre- to post-intervention changes in grams/day and frequency of consumption of de-promoted (i.e., discouraged) food groups, such as store-bought high-fat meats and unhealthy drinks; and 2) comparing pre- and post-intervention changes in healthy and unhealthy preparation methods.

## Materials and methods

### Setting

This study was a quasi-experimental intervention evaluation conducted in the Kitikmeot region in NU and the Beaufort Delta region in the NWT. Two remote communities in NU along with one semi-remote and one remote community in the NWT received the HFN intervention program. Two additional remote communities (one each in NU and the NWT) served as comparison controls, receiving a delayed intervention following the completion of post-intervention data collection. These communities have previously been described in detail [13]. Communities were selected for participation because of their varying proportions of Inuit or Inuvialuit populations, socioeconomic status, degree of acculturation, and degree of traditional food access 13]. The three NU communities ranged in size from 800–1,500 people, 80-90% of whom self-identify as Inuit. The median Inuit age ranged from 20–26 years, and the employment rate ranged from 40-60%. The three communities in the NWT ranged from 400–3,500 people, with Inuvialuit populations ranging from 40-90% of the community. The median age of Inuvialuit in these communities ranged from 24–26 years, and 40-65% were employed [31]. Each of the six communities had 2–3 food stores that obtain food primarily via airplane year round, roads and/or ice roads for part of the year, and barge or sea lift once per year when the sea ice melts. Food is also obtained, to varying degrees, by traditional means (e.g. hunting, fishing, food sharing networks).

### Sampling

Households were recruited by random selection using up-to-date community housing maps provided by the local governments. One resident per household, ideally the person who was the main food shopper/preparer, was recruited. Exclusion criteria included pregnant and lactating women (due to this groups’ different nutritional requirements and possible changes in dietary habits) and residents <19 years. Assignment of communities to the intervention or control group was decided by the government and was based on population size, the percentage of Inuit or Inuvialuit in the population, wage economy and engagement in traditional food gathering practices [32].

### Intervention development and implementation

Community participatory research [24] was used to identify the themes of the interventions and have previously been described in detail [13]. The intervention program included five phases and each focused on promoting certain healthy food options and approaches to physical activity. The first phase entailed six months of informative research to understand local conceptions of healthy foods and cultural norms around food practices. In the second phase, In-depth interviews with community stakeholders and community workshops were conducted through two two-day workshops to develop the intervention [19,24]. The interviews and workshops sought information on important dietary practices and behaviors valued by Inuit and Inuvialuit community members. In the third phase, the evidence was reviewed and these results were integrated into culturally-appropriate, relevant, and attainable intervention components and materials. The fourth phase involved 3-day training for intervention trainees, local community health representatives, project coordinators, and local store staff and the fifth phase was the actual program implementation and evaluation. Elders and community members emphasized the importance of ‘country’ foods (foods from the land, air, or sea such as caribou, seal, fish, ptarmigan, goose, or berries); as such, many of the intervention materials focused on ways to prepare traditional foods. Traditional procurement practices, such as hunting, fishing, collecting ice water, and berry picking also promote physical activity, which was another important HFN intervention program component. Promotion of healthy foods and de-promotion of unhealthy foods was undertaken in grocery stores, at community sites, through posters, flyers, interactive sessions, educational displays, and through media such as radio and television announcements [13]. For example, materials were developed to promote consumption of water in place of carbonated beverages and posters displayed nutrient content comparisons between traditional meat such as Arctic char and caribou versus processed meat in grocery stores [28]. Program implementation strategies included healthy breakfasts, healthy meal planning and cooking, and education sessions on consuming sufficient amounts of vitamins and minerals, among other activities to promote healthy diet. Implementation sites included food stores, health clinics, offices, as well as at community special events, such as feasts [23]. Similar interventions were displayed through local television ads, and local radio broadcast stories featuring family activities to improve diet and exercise [32].

### Data collection

Data collection was carried out at two time points in each community: baseline and 12 months post-intervention. Baseline data were collected between June and October 2008 (summer to fall) for the NU communities and between July 2007 and July 2008 for the NWT communities [13]. Hence, the one-year intervention period commenced October 2008 and July 2008 for the NU and NWT communities, respectively [28]. Pre- and post-intervention data were collected by local community health workers, community members, and university students all of whom were trained by the principal investigator (S.S.) in questionnaire administration and anthropometry to ensure standardization. Anthropometric measurements (height and weight) were obtained in duplicate and recorded [13]. Culturally appropriate quantitative food frequency questionnaires (QFFQ) were used to assess dietary intake at both time points. These QFFQs were previously developed and validated specifically for Inuit and Inuvialuit populations and were designed to assess dietary intake in the respective communities [33,34]. Participants were asked to report frequency of consumption over a 30-day period choosing from eight categories which ranged from “never” to “two or more times per day.” Participants reported average portion size using food models to increase participants’ accuracy of quantification.

The Adult Impact Questionnaires (AIQ) determined food acquisition and preparation behaviors, as well as demographic, socioeconomic, and psychosocial factors. Community stakeholders assessed the AIQ using face validity and Cronbach’s α indicated high internal reliability. Participants were asked for the first and second most commonly used preparation methods in the past 30 days. The questionnaire has been described previously [19]. Data collectors interviewed participants in their homes and the majority of interviews were conducted in English. Participants whose primary language was not English were interviewed by an interviewer fluent in the local language or by an interviewer aided by an interpreter. Institutional Review Board approval was obtained from the Committee on Research Ethics at the Cancer Research Centre in Hawaii and the University of North Carolina at Chapel Hill. The NU Research Institute, the Ethics Committee of the Beaufort Delta Health and Social Services Authority and the Aurora Research Institute in the NWT all provided research licenses. Informed written consent was obtained from all study participants. Participants were reimbursed for their time with CAD $25 gift cards for use at local stores.

### Outcome measures

The evaluation examined two primary outcome measures: i) consumption of de-promoted foods and ii) changes in food preparation methods. De-promoted food groups (i.e., for which consumption was discouraged) are described in Table 1 and included: high-fat meats; high-fat dairy; refined grains; high sugar drinks; unhealthy snacks; and unhealthy additions (such as high fat powdered creamer added to coffee).

**Table 1:** De-promoted food groups

**Category**	**Food items**
High-fat meats	Beef hamburger, chicken nuggets, fried chicken, lunch meat including klik and corned beef, pepperoni sticks, fish battered and fried^*^, hot dogs, and sausage or wieners.
High-fat dairy	Whole milk, carnation, cream, and half and half.
Refined grains	Fried bannock, white bread, sweet cereals including frosted flakes, and honey nut cheerios.
Unhealthy drinks	Regular pop, sweetened juice, sweetened drink including Tang, fruit punch, and kool-aid.
Unhealthy snacks	All chips, cheese curls, and regular popcorn^†^.
Unhealthy additions	Regular coffeemate, sugar or honey, regular salad dressing^†^, regular butter, margarine, lard, and mayo^†^.

^*^Nunavut.

^†^Northwest Territories.

The number of times a participant reported using a given preparation method most-often or second-most-often to prepare food (in the past 30 days) were summed for pre- and post-intervention scores to assess the intervention impact on food preparation methods. Preparation methods that reduced or did not change the fat content of prepared foods were classified as healthy. These methods included: pan fried in own fat or water; pan fried in own fat or water and drained; pan fried in own fat, drained, and rinsed; cooked with cooking spray only; microwaved, baked, roasted, or broiled without added fat; grilled; boiled; cooked with a slow cooker; boiled and drained or skimmed; steamed; smoked; raw (or frozen raw); and dried. Preparation methods that increased fat content and were classified as unhealthy included: deep-fried in oil, lard, animal fat, or shortening; pan fried in oil, lard, animal fat, or shortening; and microwaved, baked, roasted, or broiled with added fat.

### Data analysis

Baseline differences in demographic and socioeconomic variables between communities by intervention assignment were analyzed using the Student’s *t*-test for continuous normally distributed variable and the χ^2^ test for categorical variables.

The NU and NWT data were combined and individual food intake data were placed into their respective food group categories. To compare control and intervention communities’ pre- and post-intervention dietary behavior, the mean and standard deviation (SD) of total intake (gram/day), portion weight (gram/day), and frequency of intake (times/day) of de-promoted food groups were calculated for each control and intervention group independently at both time points. Dietary outcome measures included pre- and post-intervention changes to intake of each food group (total intake, portion weight, frequency of intake) and were determined using the following formula: ∆ Change = [mean (post – pre in intervention group)] – [mean (post – pre in control group)]. A positive change indicated a larger pre- to post-intervention change in intervention communities than in control communities. Since the pre- to post-intervention changes in food group intake were normally distributed, a Student’s *t*-test was used to compare the intervention and control communities. Significance of pre- to post-intervention changes within a given community for each intervention and control communities were determined using a paired t-test for normally distributed variables or a Wilcoxon Signed Rank Sum Test for non-normally distributed variables. The impact of the intervention was assessed using analysis of covariance (ANCOVA) as the suggested method for comparing before and after experimental studies [35]. For these analyses we adjusted for the age, sex, BMI, smoking, education, MSL life score, percent of people living in households with income support, and percent of the family member employed. Data were analyzed using SAS statistical software, version 9.3 (SAS Institute, Inc., Cary, NC). All tests and p-values were two-sided and considered statistically significant at α ≤ 0.05.

## Results

A total of 441 QFFQs and 494 AIQs were collected at baseline; response rates ranged from 74-93% in NU and from 65-85% in the NWT communities. Only participants who completed both pre- and post-intervention QFFQs (n = 332), and pre- and post-intervention AIQs (n = 378) were included in the analyses. Table 2 describes the 15 most commonly used preparation methods for eight foods: bannock, chicken, pork or beef, Arctic char (fish), seal, muskox or caribou, potatoes, and eggs.

**Table 2:** **Adult Impact Questionnaire (AIQ) (*Food preparation methods section)***

	**Cooking method**		
	**#1**	**#2**	
Food	First most used methods	Second most used method	Cooking method options
Bannock			1 = Did not cook in last 30 days
Chicken			2 = Deep-fried in oil, lard, animal fat, or shortening
Pork or beef			3 = Pan fried in oil, lard, animal fat, or shortening
Fish (Arctic char)			4 = Pan fried in own fat or water
Seal			5 = Pan fried in own fat or water and drained
Muskox or caribou			6 = Pan fried in own fat, drained, and rinsed
Potatoes			7 = Cooked with cooking spray only
Eggs			8 = Microwaved, baked, roasted, broiled without added fat
			9 = Microwaved, baked, roasted, broiled with added fat
			10 = Grilled
			11 = Boiled, cooked with a slow cooker
			12 = Boiled and drained or skimmed
			13 = Steamed
			14 = Smoked
			15 = Raw (or frozen raw)
			16 = Dried
			17 = Other
			18 = No other method

Now I am going to ask you about how your household usually prepares different foods.

● Please think about how the foods listed here were cooked at home IN THE PAST 30 DAYS.

● How did you most often cook [food name] (Method #1) in the past 30 days?

● Now tell me how you next most often cooked [food name] (Method #2).

● Please refer to Part 3 on the answer sheet for response choices.

Table 3 describes the demographic characteristics of Inuit/Inuvialuit men and women by intervention assignment. Compared to the control communities (n = 111), participants in the intervention communities (n = 221) were significantly older (*p* = 0.01) and were less likely to have at least one household member on income support (*p* = 0.03), but were similar in all other demographic variables.

**Table 3:** Characteristics of the study sample by intervention assignment

	**Intervention**		**Control**		**p-value**
	**(n = 221)**		**(n = 111)**		
	**Mean**	**SD**	**Mean**	**SD**	
**Age (years)**	45.5	14.1	41.9	10.7	0.01^1^
	**N**	**%**	**n**	**%**	
**Gender**					
Men	44	20	20	18	
Women	177	80	91	82	0.68^2^
**Material Style of Life (MSL)**					
Low (MSL score <8)	64	30	28	25	
Intermediate (MSL score 8–12)	77	36	35	32	
High (MSL score >12)	73	34	47	43	0.31^2^
**Education**^3^					
Low	81	38	46	42	
Intermediate	87	41	39	36	
High	45	21	24	22	0.66^2^
**Number of adults living in the household who receive income support**					
No	56	26	24	22	
Yes	159	74	85	78	0.03^2^
**People in household working**					
No	130	60	52	48	
Yes	85	40	57	52	0.43^2^

^1^A Student t-test was performed.

^2^A Chi-square test was performed.

^3^Low: none, some elementary school, elementary school completed, some junior high school; Intermediate:, some high school, junior high school or high school completed; High: some college or trade school, college or trade school completed, some university or university completed.

Changes in frequency (times/day), total intake (g/day) and portion sizes (g/day) of food intake between and within intervention and control groups are presented in Table 4. The frequency of high-fat meat consumption significantly decreased in the intervention communities (∆ = −0.2 times/day) and remained constant in the control communities. There was a significant decrease in the frequency of high-fat dairy product consumption in the intervention communities from 0.2 to 0.1 times/day (∆ = −0.1 times/day); in contrast, the frequency of high-fat dairy product consumption in the control communities increased from 0.1 to 0.2 times/day. The control communities had a significant increase in the frequency of refined grain product consumption (0.9 to 1.2 times/day) and unhealthy drinks (1.3 to 1.6 times/day). Within both treatment groups there was a significant increase in frequency of unhealthy additions intakes; however, the change was not statistically significant between intervention and control groups.

**Table 4:** Change in frequency, total intake and portion size of consumption of de-promoted food groups by intervention assignment among adult Inuit and Inuvialuit

**De-promoted food groups**	**Intervention**						**Control**						**Δ intervention vs. Δ control**^**2**^
	**Pre (n = 221)**		**Post (n = 221)**		**∆**^**1**^		**Pre (n = 111)**		**Post (n = 111)**		**∆**^**1**^		
	**Mean**	**SD**	**Mean**	**SD**	**Mean**	**SD**	**Mean**	**SD**	**Mean**	**SD**	**Mean**	**SD**	
**Frequency (time/day)**													
High-fat meats	0.4	0.5	0.3	0.3	**−0.2**^**†**^	0.4	0.3	0.3	0.3	0.4	0.0	0.4	**−0.2**^**†**^
High-fat dairy products	0.2	0.4	0.1	0.4	−0.1^*^	0.5	0.1	0.3	0.2	0.4	0.1^*^	0.5	**−0.2**^**‡**^
Refined grain products	0.8	0.7	0.9	0.8	0.0	0.8	0.9	0.6	1.2	1.0	**0.3**^**†**^	1.0	−0.3*
Unhealthy drinks	1.3	1.0	1.3	1.1	0.0	1.1	1.3	1.1	1.6	1.1	0.3^*^	1.2	−0.3*
Unhealthy snacks	0.3	0.5	0.3	0.4	0.0	0.4	0.3	0.4	0.3	0.3	0.0	0.4	−0.0
Unhealthy additions	2.0	1.5	2.3	1.5	0.3^*^	1.4	2.0	1.4	2.4	1.5	**0.4**^**‡**^	1.4	−0.1
**Total Intake (g/day)**													
High-fat meats	46	72	27	47	**−19.0**^**†**^	66.8	24	24	33	73	8.9	69.6	**−27.9**^**‡**^
High-fat dairy products	19	88	11	41	−8.0	91.3	6	17	18	52	11.8^*^	50.7	−19.8*
Refined grain products	69	67	69	77	0.7	85.9	110	127	112	132	2.3	150.5	−1.6
Unhealthy drinks	754	885	587	846	−166.6^*^	808.2	727	785	749	966	21.9	1028.3	−188.5
Unhealthy snacks^**3**^	49	156	17	24	−31.5^*^	153.9	36	70	17	21	−18.6^*^	68.8	−12.9^3^
Unhealthy additions	48	52	37	40	**−11.1**^**‡**^	47.3	45	55	33	46	−12.1^*^	53.8	0.9
**Portion size (g/day)**													
High-fat meats	**281**^†^	199	**195**^†^	161	−85.6	196.8	232	168	219	244	−12.6	244.0	−73.0*
High-fat dairy products	35	131	26	76	−8.3	142.6	28	81	42	92	14	114.0	−22.3
Refined grain products	204*	136	178*	110	−26.6	155.9	**274**‡	186	**216**‡	116	−57.6	181.7	31.0
Unhealthy drinks	**944**^†^	829	**723**^†^	615	−221.2	703.7	946*	639	778*	511	−168.4	625.9	−52.8
Unhealthy snacks^**3**^	**106**^†^	129	**58**^†^	41	−48.3	127.7	**138**^†^	109	**65**^†^	36	−73.8	116.6	25.5
Unhealthy additions	**56**^†^	52	**42**^†^	48	−13.9	49.7	56*	59	40*	50	−16.3	54.3	2.4

^1^Δ Change between post and pre intervention in intervention or control group = [mean (post – pre)].

^2^Δ Change intervention vs. Δ control = [mean (post – pre in intervention group)] – [mean (post – pre in control group)].

^3^A Wilcoxon Signed Rank Sum Test was performed.

A t- test was performed on all other p-values.

* p ≤ 0.05; ‡ p ≤ 0.001; ^†^p ≤ 0.0001.

Total intake of high-fat meat significantly decreased in the intervention group from 46 to 27 g/day and increased in the control group from 24 to 33 g/day (∆ = −27.9 g/day) but this was not significant statistically. Compared to the control group the portion size of high-fat meat intake decreased significantly in the intervention group (∆ = −73.0 g/day). The decrease in high-fat meats can, in part, be attributed to the significant change in processed beef or pork total intake within intervention groups (−16.8 g/day, data not shown). There was a non-significant decrease (19 to 11 g/day) in total intake of high-fat dairy products in the intervention communities, while intake significantly increased in the control communities from 6 to 18 g/day (∆ = −19.8 g/day). Unhealthy drinks significantly decreased within the intervention group from 754 to 587 g/day. Unhealthy snacks and additions significantly decreased within both the intervention and control groups, however there was no significant difference between the intervention and control groups (Table 4).

In fully adjusted ANCOVA analysis, receiving the intervention was significantly inversely associated with daily de-promoted grain intake (β = −26, 95% CI: −46, −6). A male gender was associated with a higher intake of high-fat meats (β = 20, 95% CI: 7, 33) and de-promoted grains (β = 42, 95% CI: 17, 67). Participants with the highest MSL score compared with the reference group had a lower de-promoted grain intake (β = −30, 95% CI: −57, −3). Every 10 year increase in age was associated to 112 grams of less de-promoted drink consumption (95% CI: −186, −39). People living in households with income support and/or family member employed had a lower intake of de-promoted additions than people without supports (β = −14, 95% CI: −24, −5).

Table 5 shows pre- to post-intervention changes for the most commonly used healthy and unhealthy food preparation methods between and within intervention assignment groups.

**Table 5:** Change in the most frequently reported preparation methods pre- and post- intervention by intervention assignment among adult Inuit and Inuvialuit

**Preparation methods**	**Intervention**				**Control**				**Δ intervention vs. Δ control**^**1**^
	**Pre (n = 246)**		**Post (n = 246)**		**Pre (n = 132)**		**Post (n = 132)**		
	**Mean**	**SD**	**Mean**	**SD**	**Mean**	**SD**	**Mean**	**SD**	
Unhealthy methods^2^	**2.0**^†^	1.0	**1.6**^†^	0.9	1.9	1.1	1.8	1.0	−0.2
Healthy methods^3^	**3.9**‡	1.3	**4.3**‡	1.3	4.5	1.5	4.3	1.4	**0.5**‡
***Select preparation methods***									
Microwaved, baked, roasted, broiled (**no** added fat) ^2^	2.0*	1.6	2.2*	1.4	2.3	1.6	2.0	1.3	0.5*
Microwaved, baked, roasted, broiled (added fat) ^2^	0.7*	1.4	0.4*	0.7	**0.1**^†^	0.4	**0.4**^†^	0.7	**−0.7**^†^
Deep fried in oil, lard, animal fat, or shortening^2^	**0.3**^†^	0.7	**0.1**^†^	0.3	**0.9**^†^	1.4	**0.2**^†^	0.4	**0.5**^†^
Pan fried in oil, lard, animal fat, or shortening^2^	**2.6**‡	1.7	**2.1**‡	0.3	2.6*	1.8	2.2*	1.4	−0.1*
Pan fried in own fat or water and drained; rinsed^4^	0.1*	0.3	0.2*	0.5	0.2	0.6	0.2	0.5	0.2*
Raw (or frozen raw), dried^4^	0.7*	0.9	0.8*	0.9	0.8	1.0	0.7	0.8	0.2

^1^Change in number of times method reported by individuals = (post – pre-intervention) - (post – pre-control).

^2^Wilcoxon Signed Rank Sum Test to test the intra-group difference between pre- and post- intervention.

^3^Paired t-test to test the intra-group difference between pre- and post- intervention.

^4^Two-sample t-test with equal variances.

*p ≤ 0.05; ‡ p ≤ 0.001; ^†^p ≤ 0.0001.

Healthy preparation methods increased significantly in the intervention group from 3.9 to 4.3 times/day, and there was a significant pre- to post-intervention change between groups (∆ = 0.5 times/day). Unhealthy preparation methods decreased within the intervention group from 2.0 to 1.6 times/day (p ≤ 0.0001). Pre- to post-intervention changes between the intervention and control groups included: microwaved, baked, roasted, broiled with *no* added fat (∆ = 0.5 times/day, p ≤ 0.05); pan fried in own fat or water and drained (and/or rinsed) (∆ = 0.2, p ≤ 0.05); microwaved, baked, roasted, broiled *with* added fat (∆ = −0.7 times/day, p ≤ 0.0001); and pan fried in oil, lard, animal fat, or shortening (∆ = −0.1 times/day, p ≤ 0.05). The intervention group had a significant decrease in food preparation through deep frying in oil, lard, animal fat or shortening, but the control group had a greater decrease in this preparation method (∆ = 0.5, p ≤ 0.0001).

## Discussion

Nutrition intervention programs may be beneficial for Inuit and Inuvialuit populations, which have an estimated threefold higher prevalence of heart disease compared to the Canadian national average [19,36] and increased risk factors for diabetes, obesity, and hypertension [37]. It is well established that decreasing animal fats, including high-fat dairy products and partially hydrogenated fats, aids in the reduction and prevention of obesity and its related comorbidities [38,39]. Evidence also strongly supports an inverse relationship between the consumption of fruit and vegetables and risk of several cancers, heart disease, and overall mortality [40]. This may be due to the naturally occurring essential nutrients (e.g. antioxidants, fiber, and folic acid) within fruit and vegetables [38,41]. Thus, the year-long pilot HFN intervention was designed in part to reduce reliance on high fat, high sugar, non-nutrient-dense foods and beverages and unhealthy preparation methods that added fat, and to increase utilization of healthier cooking methods, in an attempt to reduce chronic disease risk. The results of the intervention were successful in reducing the consumption of de-promoted foods and in the utilization of unhealthy cooking. There was a significant increase in the use of healthy preparation methods within 12 months. The pre-intervention evaluation of this population indicated that pan-frying with fat was one of the most frequently reported methods of preparation [18,19]. Post-intervention results from the intervention communities indicated a decrease in the use of this method and a concurrent increase in the use of pan-frying methods that did not add fat, thereby reducing added fat consumption in the population under intervention. Several epidemiological studies suggest that the consumption of fried, boiled or roasted red meat is associated with the development of cancer; it has been proposed that heterocyclic aromatic amines, potent mutagens present at ng/kg levels in cooked foods play an important role in the aetiology of human cancer [42,43]. Therefore, avoiding high-temperature cooking methods may lower the risk of cancer.

Compared to the control group, the intervention group had a greater reduction in intake of de-promoted high-fat meats, high-fat dairy, refined grain products, and unhealthy drinks, all of which are commonly consumed food groups in this population [11,12,27,29]. Baseline studies determined that sweetened juices/drinks made the largest contribution to energy, carbohydrate, and sugar in NU and the first and second largest contribution in the NWT. Regular soft drinks and white bread were also top contributors to energy, carbohydrate, and sugar for both populations. Furthermore, butter, margarine, lard, and high-fat meats, including sausages and lunchmeats, were the top contributors to fat [16,17,27,29]. The reduced consumption of de-promoted food groups (particularly refined grains, unhealthy drinks, high-fat dairy products and high-fat meats) in the intervention group compared to control could explain the decreases in energy intake (average of 317 kcal/day), protein intake (21 g/day), carbohydrate intake (37 g/day), and overall Body Mass Index (BMI) (p = 0.002) [44]. Improved intake of vitamin A and D were also observed. These nutrients are naturally abundant in the traditional foods consumed by Arctic Indigenous populations [10,13,45]. Therefore, it may be inferred that dietary adequacy improved, in part, as a result of the observed significant increase in traditional food intake (from 1.4 to 1.7 times/day within the intervention group).

To our knowledge, there have been no studies on the impact of interventions within Inuit/Inuvialuit populations; therefore, the effectiveness of HFN’s community-based program must be compared with interventions targeting other Indigenous and/or remote populations. A recent review on the community-based interventions in prepared-food sources found some promising results however the outcome measures were limited [46]. Many of the interventions included in this review were not formal studies but rather certification or campaign programs operated by local health departments. Therefore, the voluntary nature of the programs may explain why they varied in levels of reach. Similar to the present study, a store-based intervention targeting Native American adults living on Arizona reservations saw no change in the consumption of high-sugar, high-fat snacks and fast food. They found that the consumption of the comparison group increased significantly for less healthy foods over the year of the intervention program, which may indicate that in general, people are eating less healthy. It is possible that the program helped keep the intervention group’s diets from getting unhealthier [47].

A family-based intervention conducted with the Six Nations Reserve in Ohsweken, Ontario made similar observations [48]. They reported a decrease in intake of fatty foods, oils, and sodas paralleling HFN’s decrease in high-fat meat consumption, unhealthy drinks, and unhealthy cooking methods. However, some interventions among Indigenous populations outside of North America have shown promising results. Promotion of local foods and a traditional diet have resulted in increased intake of local accessible foods as well as increased nutrient intake in Indigenous populations in Micronesia [45], the Dalit in India [49], and Australia [50].

HFN was a community-based and community-driven intervention project. Community interventions have much greater potential to reduce weight and related health risks than individual weight loss programs [48,51]. There is greater possibility for sustainability if the programs partner with community-based institutions such as schools and stores [52]. However, it is important to consider the remoteness of these Arctic populations and the economic and environmental barriers that limit the feasibility of an active lifestyle and access to fresh nutritious foods. Future program development should focus on mitigating these barriers by improving the accessibility and affordability of healthy foods (e.g. fruit and vegetables and low-fat, low-sugar store bought items); furthermore, traditional foods high in protein, iron, and vitamins should be promoted [10,16,17,53]. Marine omega-3 fatty acids, contained in Arctic char and other fish and marine mammals, have proven protective effects against coronary heart disease in several diverse populations [54]. Continued efforts to revitalize traditional food systems, such as hunting, gathering, and food-sharing, are equally important as they have a multitude of health and well-being benefits. Ongoing trials with longer intervention periods and larger sample populations are needed to monitor HFN’s impact on chronic disease risk.

### Strengths and limitations

The sample was predominantly female (80-82%) because the study targeted the primary food shoppers and preparers. Bias may also have been introduced by the lower response rates that were observed for some communities. Given the potential for variation in access to store bought and traditional foods throughout the year, differences in the time of year for collection of baseline and follow up data, particularly for the NWT communities, may also have led to bias. In addition, limited data were available for potential confounders. However, baseline dietary differences between control and intervention groups were unchanged when stratified analyses were examined for age and income support (variables that were differentially distributed among the control and intervention groups). It is unlikely that the control groups were exposed to the intervention content that was disseminated via television and radio, as access to media between communities is limited in this remote region. Therefore, results may not be generalizable to male Inuit and Inuvialuit populations. Recall bias, which may occur with QFFQs, is another potential limitation [55]. However, validation studies of the QFFQs used in this study confirmed relative agreement with multiple 24-hour recalls in this population [33,34].

This study provides the first data on the impact of a multi-institutional, community-based nutrition intervention program among Inuit in NU and Inuvialuit in the NWT. These data will contribute not only to the limited literature, but may also contribute to government policy decision-making related to Inuit and Inuvialuit nutrition and health. The data collection instruments are current and culturally relevant for this population.

## Conclusion

The results from this study demonstrate that the HFN program was effective for reducing consumption of high fat, high sugar foods and beverages of low nutritional density and reliance on preparation methods that add fat to foods. These findings may be considered for other interventions with Indigenous populations living in other remote areas worldwide.

## Abbreviations

NU: Nunavut; NWT: Northwest Territories; HFN: Healthy Foods North; QFFQ: Quantitative food frequency questionnaire; AIQ: Adult Impact Questionnaire; MSL: Material Style of Life; IEAS: Intervention Exposure Assessment Survey.

## Competing interests

The authors declare they have no competing interests.

## Authors’ contributions

FK conducted the data analysis, drafted and finalized the manuscript. MP drafted the manuscript. EM and LB initiated and implemented the intervention in NWT and Nunavut and oversaw all field activities. AC critically reviewed the manuscript. SS developed the designed the study, trained data collectors, and oversaw data collection. All authors were responsible for interpretation of the data and critically reviewed its content and have approved the final version submitted for publication.
